# Association of coffee intake with risk of all-cause and colorectal cancer-specific mortality among individuals with colorectal cancer in the Multiethnic Cohort Study

**DOI:** 10.1007/s10552-026-02246-w

**Published:** 2026-09-09

**Authors:** Kathryn R. K. Benson, Irina Tolstykh, June M. Chan, Sorbarikor Piawah, Lynne R. Wilkens, Song-Yi Park, Stacey A. Kenfield, Loïc Le Marchand, Christopher A. Haiman, Iona Cheng, Erin L. Van Blarigan

**Affiliations:** 1 Department of Medicine, University of California, San Francisco, CA USA; 2 Department of Epidemiology and Biostatistics, University of California, 550 16th St., San Francisco, CA 94158 USA; 3 Department of Urology, University of California, San Francisco, CA USA; 4 Population Sciences in the Pacific Program, University of Hawai’i Cancer Center, University of Hawai’i; Honolulu, Honolulu, Hawai’i USA; 5 Department of Population and Public Health Sciences, Keck School of Medicine, University of Southern California, Los Angeles, CA USA

**Keywords:** Caffeine, Cancer survival, Nutrition, Colon, Rectal

## Abstract

**Purpose:**

Coffee may influence colorectal cancer (CRC) survival, but prior studies had limited generalizability.

**Methods:**

We analyzed 1,098 participants from the Multiethnic Cohort study diagnosed with CRC between 1993–2008 who completed a food frequency questionnaire after diagnosis and were followed through 2019. Multivariable Cox proportional hazards regression models were used to examine coffee (total, caffeinated, decaffeinated) in relation to all-cause and CRC-specific mortality. Secondarily, we examined caffeine intake and explored associations by race and ethnicity.

**Results:**

Overall, coffee intake was not associated with risk of mortality. However, decaffeinated coffee was associated with lower risk of all-cause mortality (HR ≥1 vs. 0 cups/day: 0.71; 95% CI [0.55 – 0.91]). Additionally, associations were observed between total and caffeinated coffee in relation to all-cause mortality among Native Hawaiian participants (HR per 1 cup/day: 0.62 [0.41 – 0.94] and 0.55 [0.31 – 0.97], respectively); and with CRC-specific mortality among White participants (HR per 1 cup/day: 0.48 [0.25–0.94] and 0.45 [0.23 – 0.90], respectively). In the total cohort, caffeine intake was associated with 22% lower risk of all-cause mortality (HR 4th quartile vs. 1st quartile: 0.78 [0.62—0.99]) and 52% lower risk of CRC-specific mortality (HR 0.48 [0.27—0.87]).

**Conclusions:**

Overall, coffee intake after CRC diagnosis was not associated with all-cause or CRC-specific mortality. Decaffeinated coffee was associated with lower risk of all-cause mortality, and caffeine was associated with lower risk of all-cause and CRC-specific mortality.

**Supplementary Information:**

The online version contains supplementary material available at https://doi.org/10.1007/s10552-026-02246-w.

## Background

Colorectal cancer (CRC) is the third most common cancer worldwide [1] and the second leading cause of cancer death in the United States [2]. Given the high rates of CRC mortality, it is important to understand the impact of modifiable factors on CRC pathogenesis and progression. Coffee specifically has been identified as a potential factor that may influence outcomes among people with CRC. In vitro and in vivo studies have found coffee to be antiproliferative, pro-apoptotic, antioxidant, and anti-inflammatory [3, 4]. Studies suggest that coffee may alter the gut microbiome and increase insulin sensitivity, which are other mechanisms by which coffee may be protective against CRC incidence and proliferation [5–7].

There is growing evidence of an association between coffee intake and lower risk of CRC-specific and all-cause mortality [8–12]. Among 953 individuals with stage III colon cancer, consumption of 2–3 cups or ≥4 cups per day of total coffee or caffeinated coffee (compared to no coffee intake) was associated with longer disease-free survival [8]. Among 1,599 patients with stage I-III CRC, drinking 4 or more cups per day of total coffee was associated with lower risk of CRC-specific and all-cause mortality [9]. A systematic review and meta-analysis examining these findings reported “limited-suggestive” evidence for the positive impact of post-diagnosis coffee intake on all-cause mortality among individuals with CRC [13]. A more recent study among 1719 participants with stage I-III CRC in the Netherlands reported a U-shaped association between coffee consumption and risk of mortality; with the lowest risk of all-cause mortality seen at 4 cups/day [14]. An important limitation of the studies to date, however, is limited racial and ethnic diversity in the study populations [8–10].

In the United States, there are well documented racial and ethnic disparities in CRC incidence and mortality [2]. Compared to non-Hispanic White counterparts, Black males and females have 40% and 24% higher rates of CRC mortality, respectively [2]. Conversely, data suggest Hispanic and Asian American/Pacific Islander populations have lower risk of CRC mortality compared to their White counterparts [2]. However, disaggregated data have demonstrated higher rates of CRC among Japanese and Native Hawaiian people compared to White people [15, 16]. Based on national data in the United States, coffee intake varies across racial and ethnic groups. On average, non-Hispanic White individuals consume an average of 12.4 oz (approximately 1.5 cups) of coffee per day compared to 8.3 oz (approximately 1 cup) per day among Hispanic individuals and 4.8 oz (0.5 cup) among Black individuals [17]. Thus, we aimed to understand whether coffee intake is associated with risk of all-cause or CRC-specific mortality in a racially and ethnically diverse sample of individuals with CRC.

## Methods

### Study population

This prospective analysis examined participants from the Multiethnic Cohort (MEC) study diagnosed with CRC after entry in the cohort and prior to the repeat diet assessment. The study design for the MEC has been described in detail elsewhere [18]. Relevant to this analysis, participants in the MEC completed a comprehensive survey at enrollment in 1993–1996 that included a validated food frequency questionnaire (FFQ) [19]. The FFQ was repeated as part of the 10-year follow-up in 2003–2008 [20]. Both surveys are publicly available online through the University of Hawai’i Cancer MEC Study website [21]. Incident cases of CRC are identified in the MEC through annual linkage with the Los Angeles County Cancer Surveillance Program, the California Cancer Registry, and the Hawaii Tumor Registry, which are all part of the National Cancer Institute’s Surveillance, Epidemiology and End Results (SEER) Program.

To be eligible for this analysis, participants must have been free of CRC at the time of cohort enrollment and diagnosed with CRC prior to the follow-up FFQ survey (*n*=2,433). Subsequently, participants who did not complete the follow-up questionnaire (*n*=524) or who died prior to completing the follow-up questionnaire (*n*=735) were excluded, leaving 1,174 participants. Finally, 58 participants were excluded due to missing or implausible FFQ data, 13 were excluded due to missing CRC disease stage at diagnosis, and 5 were excluded due to short follow-up (less than 3 months) after the follow-up FFQ, leaving 1,098 participants eligible for analysis. Prior work examined a similar final cohort and reported demographic and health behavior differences among the initial participants diagnosed with CRC and the final included participants (after excluding those who died prior to follow-up and with missing survey data) [22]. Excluded participants were more likely to have distant disease and less than high school education; additionally, there was a lower proportion of Black and Latino participants in the final sample compared to the initial sample [22]. When examining change in coffee consumption from pre- to post CRC diagnosis, an additional 19 participants were excluded because they were missing the baseline FFQ.

### Assessment of coffee intake and caffeine intake

Coffee intake was assessed at enrollment (1993–1996) and at one follow-up (2003–2008) using the FFQ. The primary analyses of this study utilized the survey data collected from 2003–2008 to assess post-diagnosis health behaviors. Participants were asked how often they consumed 1 cup or mug of “regular coffee” and “decaffeinated (decaf) coffee.” They were also asked how often they consumed 1 cup or mug of “cappuccino or café con leche (includes café au lait, café latte).” There were nine possible frequency options ranging from “never or hardly ever” to “4 or more times a day.” Prior validity studies with similar FFQs have found high reproducibility and validity with participants’ report of coffee consumption [19, 23, 24].

For consistency with prior studies, we defined our primary exposure as total coffee (caffeinated and decaffeinated). We also examined caffeinated coffee and decaffeinated coffee separately along with total caffeine intake (regardless of source). Sources of caffeine in this sample included coffee, black tea, green tea, regular soda, and diet soda. Coffee intake was analyzed as a continuous variable and as a discrete variable. The categorical groups of coffee were 0 cups, > 0 to <1 cups, 1 to <2 cups, and ≥ 2 cups/day. These cut-points were chosen to obtain similar sized groups based on the distribution of coffee intake within the sample. In a secondary analysis, we included cappuccino in the estimation of total coffee intake and found similar results to the main analysis (**Supplemental Table** 1).

**Table 1:** Characteristics of participants in the analytic sample by total coffee intake (caffeinated and decaffeinated)

	Total	Total post-diagnosis coffee intake (cups/day)			
		None	>0 to <1	1 to <2	≥2
*N*^a^	1098	191	293	400	214
Age at diagnosis, mean (SD)	68.8 (8.3)	69.1 (8.7)	68.6 (8.3)	69.3 (8.2)	67.8 (8.0)
Years between diagnosis and follow-up survey, mean (SD)	5.0 (3.4)	4.8 (3.4)	5.2 (3.3)	4.9 (3.3)	5.0 (3.4)
Sex, *n* (%)					
Female	495 (45)	98 (51)	137 (47)	173 (43)	87 (41)
Male	603 (55)	93 (49)	156 (53)	227 (57)	127 (59)
Race and ethnicity, *n* (%)					
Black or African American	156 (14)	41 (22)	49 (17)	44 (11)	22 (10)
Japanese American	457 (42)	73 (38)	127 (43)	169 (42)	88 (41)
Latino	173 (16)	22 (12)	48 (16)	70 (18)	33 (15)
Native Hawaiian	81 (7)	16 (8)	19 (7)	29 (7)	17 (8)
White	231 (21)	39 (20)	50 (17)	88 (22)	54 (25)
Tumor location, *n* (%)					
Colon	804 (73)	144 (75)	221 (75)	293 (73)	146 (68)
Rectal/Both	294 (27)	47 (25)	72 (25)	107 (27)	68 (32)
Stage at diagnosis, *n* (%)					
Localized	658 (60)	107 (56)	177 (60)	237 (59)	137 (64)
Regional	397 (36)	72 (38)	106 (36)	150 (38)	69 (32)
Distant	43 (4)	12 (6)	10 (3)	13 (3)	8 (4)
Treatment, *n* (%)^b^					
Surgery	1062 (97)	182 (95)	284 (97)	388 (97)	208 (97)
Radiation	85 (8)	18 (9)	14 (5)	36 (9)	17 (8)
Chemotherapy	307 (28)	66 (35)	74 (25)	115 (29)	52 (24)
Education, *n* (%)					
High school or less	457 (42)	74 (39)	111 (38)	183 (46)	89 (42)
Some college	350 (32)	70 (37)	88 (30)	132 (33)	60 (28)
College or greater	280 (25)	46 (24)	90 (31)	82 (21)	62 (29)
Unknown	11 (1)	1 (1)	4 (1)	3 (1)	3 (1)
Marital status, *n* (%)					
Married	663 (60)	102 (53)	173 (59)	254 (64)	134 (63)
Separated, divorced, widowed	349 (32)	72 (38)	94 (32)	123 (31)	60 (28)
Never married, unknown	88 (8)	17 (9)	26 (9)	23 (6)	20 (9)
Body mass index, kg/m^2^ mean (SD)	26.4 (5.1)	26.5 (5.3)	26.3 (5.2)	26.2 (4.7)	26.7 (5.3)
Physical activity, min/wk, mean (SD)	522.6 (608.3)	449.4 (504.3)	467.5 (507.9)	541.5 (606.2)	625.3 (783.2)
Energy intake, kcal/d, mean (SD)	1842.8 (818.1)	1730.4 (779.2)	1794.2 (794.0)	1868.3 (835.6)	1962.2 (838.1)
Regular soda, g/day mean (SD)	55.0 (162.4)	52.7 (146.6)	49.3 (153.7)	58.4 (155.2)	58.7 (197.6)
Diet soda, g/day mean (SD)	74.0 (208.8)	62.8 (181.7)	72.7 (214.6)	64.8 (196.5)	102.9 (241.8)
Black tea, g/day mean (SD)	40.5 (116.4)	49.3 (143.6)	46.5 (118.5)	26.1 (73.7)	51.4 (146.1)
Green and herbal tea, g/day mean (SD)	78.5 (154.2)	84.4 (185.1)	97.9 (169.3)	60.6 (118.0)	80.3 (158.9)
Omega-3 Fatty Acids intake, g/day mean (SD)	1.68 (0.84)	1.57 (0.83)	1.68 (0.85)	1.67 (0.84)	1.79 (0.83)
Caffeine, mg/day, mean (SD)	109.1 (95.9)	40.3 (54.0)	60.5 (49.6)	103.7 (45.7)	247.0 (103.9)
Alcohol use, g/day, mean (SD)	6.2 (15.3)	5.8 (19.8)	4.8 (12.0)	7.3 (15.8)	6.5 (13.6)
Smoking status, *n* (%)					
Never smoked	408 (37)	105 (55)	129 (44)	115 (29)	59 (28)
Former smoking	635 (58)	80 (42)	161 (55)	260 (65)	134 (63)
Current smoking	55 (5)	6 (3)	3 (1)	25 (6)	21 (10)
Smoking pack year history	15.1 (19.2)	10.0 (17.1)	11.4 (16.9)	17.2 (19.8)	20.5 (20.9)
Other cancer diagnosis (yes), *n* (%)	206 (19)	26 (14)	54 (18)	81 (20)	45 (21)
Comorbidity index, mean (SD)^c^	2.53 (1.52)	2.60 (1.41)	2.54 (1.52)	2.48 (1.56)	2.58 (1.55)

^a^Characteristics reported were collected at the follow-up survey except race and ethnicity, sex, and education (collected with the initial survey), as well as comorbidity index (method discussed in footnote below)

^b^Treatment modalities (surgery, radiation, chemotherapy) are not mutually exclusive

^c^Comorbidity index includes heart disease (acute coronary syndrome or angina), stroke, asthma, Parkinson’s disease, diabetes, kidney stones, ulcer (stomach or duodenal), rheumatoid arthritis, glaucoma, hypertension, gallstones, intestinal polyps. Specific comorbidities were reported as present if they were reported at the initial survey, the follow-up survey, or a survey conducted in between these time points

### Assessment of other covariates

Covariate data were gathered with a comprehensive 26-page self-administered survey at the time of enrollment (1993–1996) including demographics, body size, medical histories and comorbidities, medication use, family history, smoking history, physical activity, and FFQ. Follow-up surveys were administered every 4–6 years with dietary data updated on one follow-up survey (2003–2008). Energy intake and the amounts of specific foods or beverages (e.g. alcoholic beverages, red meat, fruits and vegetables) and nutrients (e.g. dietary fiber, long-chain fatty acid *n*−3, vitamin D, carbohydrates) were calculated from the FFQ, as previously described [19, 20]. Additional covariates of interest from the initial and follow-up survey data included age, sex, race and ethnicity, educational background, marital status, body mass index (BMI), physical activity, smoking behavior, SEER tumor characteristics (tumor location, stage), SEER treatment history (surgery, chemotherapy, radiation), family history of CRC, and medical comorbidities. A comorbidity index was created as a summative score of comorbidities including heart disease (acute coronary syndrome or angina), stroke, asthma, diabetes, ulcer (stomach or duodenal), rheumatoid arthritis, glaucoma, hypertension, kidney stones, Parkinson’s disease, prior cholecystectomy, and intestinal polyps. A separate variable captured comorbidity of other cancer (yes/no) in addition to CRC because history of an additional cancer was highly correlated with risk of mortality in the study population. Data on tumor site, stage, and treatment were obtained through linkage to SEER registry databases [25].

### Outcome measures

Outcome measures were all-cause mortality (primary) and CRC-specific mortality (secondary). Dates and causes of death in the MEC were obtained through linkage with California and Hawaii vital record databases, the National Death Index, and state death certificate files. For these analyses, complete case and outcome data was available through Dec. 31, 2019.

### Statistical analysis

Multivariable Cox proportional hazards regression models were used to estimate adjusted hazard ratios (HR) and 95% confidence intervals (CI) for the associations of coffee intake with all-cause and CRC-specific mortality. We explored survival curves for all primary exposure and outcome variables, which all met the proportionality assumption. Person-time was accrued from completion of the post-diagnosis FFQ until death or end of follow-up (December 31, 2019), whichever came first. When examining risk of CRC-specific mortality, deaths due to other causes were censored at time of death.

The minimally adjusted multivariable models (Model 1) were adjusted for age, sex, race and ethnicity, BMI (kg/m^2^), and years from diagnosis to survey completion. Given the MEC design with two FFQs, time from CRC diagnosis to assessment of post-diagnosis health behaviors varied and thus we adjusted for time from diagnosis to exposure assessment in all models.

For the fully adjusted multivariable models, we considered additional factors potentially associated with risk of all-cause or CRC-specific mortality. The candidate covariates included educational attainment, marital status, comorbidity index, other cancer diagnosis, family history of CRC, physical activity, smoking history, dietary factors (calories, carbohydrates, meat, fiber, fruits and vegetables, alcohol, omega-3 fatty acids, nuts, sugary beverages, regular soda, diet soda, green tea, black tea), tumor characteristics (tumor location, SEER stage), and treatment history (surgery, chemotherapy, radiation). We examined each of these factors in relation to our primary outcome (all-cause mortality); variables that were associated with all-cause mortality with *p*-value < 0.2 were included in an initial multivariable model. To build a more parsimonious model, we subsequently excluded the variable with the largest *p*-value. If exclusion of the variable changed the HR for our main exposure by 10% or more, the variable was retained in the model. Following this model building process, the covariates included in the fully adjusted multivariable model (Model 2) included factors in Model 1 plus cancer site (colon vs rectum), SEER stage, educational attainment (high school or less, some college, vs college or greater), marital status (married, separated, divorced, widowed, never married), smoking status (never, former, current), physical activity (min/week), comorbidity index, history of another cancer (yes vs no), alcohol intake (g/day), and omega-3 fatty acid intake (g/day). To assess the independent effects of caffeinated and decaffeinated coffee, Model 3 was mutually adjusted for caffeinated and decaffeinated coffee. For participants who were missing covariate data on the follow-up survey (after CRC diagnosis) for BMI (*n*=64), smoking status (*n*=37), educational attainment (*n*=11) or marital status (*n*=34), we carried forward these data from the initial survey (prior to CRC diagnosis). If a participant was missing physical activity data (*n*=23) at the follow-up time point, it was coded using a missing indicator. We performed a sensitivity analysis excluding all records with missing data and the results were not meaningfully changed.

We conducted several secondary analyses. First, to examine whether our results varied by race and ethnicity, we ran models stratified by racial and ethnic group (Black, Japanese, Latino, Hawaiian, and White) and assessed for statistical heterogeneity by race and ethnicity via an interaction term with continuous coffee intake and race/ethnicity using wald chi-square tests. Second, we assessed the association between caffeine intake from any source (coffee, black tea, green tea, regular soda, diet soda) and risk of all-cause and CRC-specific mortality. Third, we examined change in coffee intake from pre-CRC diagnosis to post-CRC diagnosis in relation to risk of CRC-specific and all-cause mortality. Change in total coffee intake was defined as maintaining <1cup/day, decreasing from ≥1 cup per day to <1 cup per day, increasing from <1 cup per day to ≥1 cup per day, and maintaining ≥1 cup per day of total coffee consumption, given 1 cup/day was the median intake at the initial and follow-up surveys.

Lastly, we conducted several sensitivity analyses to assess for potential bias. First, to assess the potential for reverse causation, we excluded participants who died within 1 year after completing the post-diagnosis FFQ and subsequently those who died within 2 years, consistent with prior studies [26]. We also conducted separate sensitivity analyses excluding participants with distant disease at diagnosis defined based on SEER staging (*n*= 43), participants who did not have surgical resection (*n*=36), and participants who did have radiation (*n*=85) to understand if differences were influenced by disease stage or treatment modalities. Finally, as mentioned above, we conducted a sensitivity analysis excluding participants (*n*=107) with missing covariate data.

## Results

This analysis included 1098 individuals diagnosed with CRC. The median time from diagnosis to the post-diagnosis survey was 4.8 years among men (IQR: 2.0, 7.6) and 4.4 years among women (IQR: 1.8, 7.4). Within the analysis, 628 events of death were observed during follow-up, of which 107 were due to CRC. The mean follow-up from the post-diagnosis FFQ to death or end of follow-up was 10.4 years (SD 4.9 years).

Baseline characteristics of the cohort overall, and by coffee intake post-diagnosis, are detailed in Table 1. The mean age at diagnosis was 69 years with majority men (55%) and individuals with colon cancer as the primary site (73%). The cohort was primarily individuals diagnosed with SEER staged localized (60%) and regional (36%) disease, with only 4% staged with distant disease. The racial and ethnic distribution of the cohort was 14% Black or African American, 42% Japanese American, 16% Latino, 7% Native Hawaiian, and 21% White. Coffee consumption varied across race and ethnicity. White participants reported the largest portion of high (≥ 2 cups per day) coffee consumption (23%), followed by Native Hawaiian (21%), Japanese American (19%), Latino (19%), and Black/African American (14%) participants. Among the higher coffee consumption groups (1 to < 2 and ≥ 2 cups per day), there was higher physical activity per week, higher caloric intake per day, and higher rates of smoking tobacco.

The associations of coffee intake with risk of all-cause and CRC-specific mortality are detailed in Table 2. There were no statistically significant associations between total or caffeinated coffee intake and risk of all-cause or CRC-specific mortality. However, higher decaffeinated coffee intake after CRC diagnosis was associated with lower risk of all-cause mortality (HR per 1 cup/day increase: 0.82 [95% CI 0.70–0.97]). This effect remained significant when controlling for caffeinated coffee as well (Model 3).

**Table 2:** Association of post-diagnosis coffee intake with risk of all-cause and CRC-specific mortality

	Post-diagnosis coffee intake (cups per day)				*p*-trend^d^	Continuous per 1 cup/day increase
	None	0—<1	1—<2	≥ 2		
Total coffee (caffeinated and decaffeinated)						
*n*	191	293	400	214		1098
*All-cause mortality*						
Events/person-years	117/1869	171/2972	223/4249	117/2296		628/11,387
Model 1 HR [95% CI]^a^	1 (ref.)	1.00 (0.79—1.27)	0.82 (0.66—1.03)	0.87 (0.67—1.13)	0.19	0.94 (0.87—1.02)
Model 2 HR [95% CI]^b^	1 (ref.)	1.06 (0.83—1.35)	0.82 (0.65—1.04)	0.85 (0.65—1.12)	0.10	0.92 (0.85—1.00)
*CRC-specific mortality*						
Events/person-years	20/1869	30/2972	37/4249	20/2296		107/11,387
Model 1 HR [95% CI]^a^	1 (ref.)	1.06 (0.60—1.87)	0.83 (0.48—1.43)	0.89 (0.48—1.66)	0.56	0.92 (0.75—1.12)
Model 2 HR [95% CI]^b^	1 (ref.)	1.12 (0.62—2.01)	0.88 (0.49—1.59)	0.91 (0.46—1.80)	0.60	0.90 (0.73—1.12)
Caffeinated coffee						
*n*	340	245	349	164		1098
*All-cause mortality*						
Events/person-years	211/3376	132/2529	196/3701	89/1781		628/11,387
Model 1 HR [95% CI]^a^	1 (ref.)	0.98 (0.79—1.22)	0.92 (0.75—1.12)	0.87 (0.68—1.12)	0.24	0.96 (0.88—1.05)
Model 2 HR [95% CI]^b^	1 (ref.)	1.09 (0.87—1.37)	0.97 (0.79—1.19)	0.92 (0.71—1.19)	0.32	0.97 (0.88—1.06)
Model 3 HR [95% CI]^c^	1 (ref.)	1.08 (0.86—1.35)	0.90 (0.73—1.11)	0.86 (0.66—1.12)	0.17	0.96 (0.87—1.05)
*CRC-specific mortality*						
Events/person-years	37/3376	21/2529	34/3701	15/1781		107/11,387
Model 1 HR [95% CI]^a^	1 (ref.)	0.76 (0.45—1.31)	0.83 (0.52—1.32)	0.75 (0.41—1.38)	0.49	0.94 (0.76—1.16)
Model 2 HR [95% CI]^b^	1 (ref.)	0.82 (0.47—1.43)	0.91 (0.56—1.50)	0.71 (0.36—1.40)	0.44	0.90 (0.72—1.13)
Model 3 HR [95% CI]^c^	1 (ref.)	0.80 (0.45—1.41)	0.93 (0.56—1.55)	0.72 (0.36—1.44)	0.45	0.90 (0.71—1.13)
Decaffeinated coffee						
	0 cups/day	0—<1 cups/day^e^	≥ 1 cups/day^e^		*p*-trend^d^	Continuous per 1 cup/day increase
*n*	746	210	142			
*All-cause mortality*						
Events/person-years	433/7627	116/2278	79/1482			628/11,387
Model 1 HR [95% CI]^a^	1 (ref.)	0.88 (0.72—1.08)	0.86 (0.67—1.09)		0.22	0.90 (0.77—1.06)
Model 2 HR [95% CI]^b^	1 (ref.)	0.89 (0.72—1.10)	0.74 (0.57—0.94)		0.02	0.82 (0.70—0.97)
Model 3 HR [95% CI]^c^	1 (ref.)	0.84 (0.68—1.05)	0.71 (0.55—0.91)		0.01	0.81 (0.69—0.96)
*CRC-specific mortality*						
Events/person-years	74/7627	20/2278	13/1482			107/11,387
Model 1 HR [95% CI]^a^	1 (ref.)	0.98 (0.60—1.62)	0.95 (0.52—1.72)		0.86	0.87 (0.57—1.33)
Model 2 HR [95% CI]^b^	1 (ref.)	1.19 (0.71—2.00)	1.03 (0.54—1.94)		0.95	0.94 (0.59—1.48)
Model 3 HR [95% CI]^c^	1 (ref.)	1.20 (0.70—2.07)	0.99 (0.51—1.89)		0.96	0.92 (0.57—1.46)

^a^Model 1 adjusted for age, sex, race and ethnicity, BMI, and years from diagnosis to survey completion

^b^Model 2 adjusted for factors in Model 1 and cancer site (colon vs rectum), stage, educational attainment, marital status, smoking, physical activity (< 60 min/week, 60–150 min/week, ≥ 150 min per week), comorbidity index (detailed above in Table 1), other cancer (dichotomous), alcohol intake (g/day), and omega 3 fatty acid intake (g/day)

^c^Model 3 includes all variables in Model 2 and caffeinated and decaffeinated coffee are mutually adjusted for one another

^d^*P*-trend assessed across categorical groups

^e^Decaffeinated coffee top categories of consumption were combined given limited sample size reporting ≥ 2 cups/day (*n* = 33)

The associations of coffee intake with risk of overall and CRC-specific morality by racial and ethnic group are shown in Table 3. While there was no statistically significant evidence of effect modification, the only statistically significant within-group associations were seen among the White and Native Hawaiian participants. Among the White participants, higher total and caffeinated coffee intake were associated with lower risks of CRC-specific mortality (HR per 1 cup/day increase: 0.48 [0.25–0.94] and 0.45 [0.23–0.90], respectively). Additionally, similar to the overall cohort, consumption of decaffeinated coffee among White participants was significantly associated with lower risk of all-cause mortality (HR per 1 cup/day increase: 0.65 [0.43–0.98]). This association was not seen among any of the other racial or ethnic subgroups. Among the Native Hawaiian participants, higher total and caffeinated coffee were associated with lower risk of all-cause mortality (HR per 1 cup/day increase: 0.62 [0.41–0.94] and 0.55 [0.31–0.97], respectively), which was not seen among any of the other racial or ethnic subgroups.

**Table 3:** Association of post-diagnosis coffee intake with risk of all-cause and CRC-specific mortality by race/ethnicity

	African American/Black	Japanese American	Latino	Native Hawaiian^b^	White	*p-value*
Total coffee, mean (SD), cups/day	0.78 (0.98)	1.00 (0.99)	1.01 (0.88)	1.03 (1.10)	1.17 (1.15)	0.0095
Caffeinated coffee, mean (SD), cups/day	0.60 (0.84)	0.79 (0.90)	0.78 (0.85)	0.73 (0.92)	0.93 (1.04)	0.015
Decaffeinated coffee, mean (SD), cups/day	0.18 (0.44)	0.21 (0.51)	0.23 (0.44)	0.30 (0.65)	0.23 (0.55)	0.47
*All-cause mortality*						*p*-interaction^c^
Events/person-years	103/1520	240/4956	100/1783	46/855	139/2274	
Total coffee HR [95% CI]^a^	0.88 (0.68—1.14)	1.04 (0.90—1.20)	0.98 (0.76—1.26)	0.62 (0.41—0.94)	0.87 (0.74—1.04)	0.33
Caffeinated coffee HR [95% CI]^a^	0.83 (0.62—1.13)	1.10 (0.95—1.27)	0.89 (0.67—1.19)	0.55 (0.31—0.97)	0.95 (0.79—1.14)	0.31
Decaffeinated coffee HR [95% CI]^a^	1.01 (0.58—1.76)	0.86 (0.66—1.13)	1.28 (0.82—1.99)	0.70 (0.39—1.25)	0.65 (0.43—0.98)	0.70
*CRC-cause mortality*						*p*-interaction^c^
Events/per person-years	19/1520	40/4956	20/1783	8/855	20/2274	
Total coffee HR [95% CI]^a^	0.89 (0.48—1.66)	1.17 (0.82—1.67)	1.03 (0.55—1.91)	-	0.48 (0.25—0.94)	0.21
Caffeinated coffee HR [95% CI]^a^	0.85 (0.42—1.73)	1.08 (0.73—1.61)	1.05 (0.54—2.03)	-	0.45 (0.23—0.90)	0.26
Decaffeinated coffee HR [95% CI]^a^	1.03 (0.33—3.15)	1.39 (0.74—2.62)	0.89 (0.18—4.34)	-	1.06 (0.38—2.96)	0.41

^a^HR [95% CI] per 1 cup/day increase adjusted for age, sex, BMI, years from diagnosis to survey, cancer site (colon vs rectum), stage, educational attainment, marital status, smoking, physical activity (< 60 min/week, 60–150 min/week, ≥ 150 min per week), comorbidity index, other cancer (dichotomous), alcohol intake (g/day), and omega 3 fatty acid intake (g/day). Models that additionally mutually adjusted for caffeinated and decaffeinated coffee are omitted for simplicity given similar findings to models displayed

^b^Insufficient number of events to run CRC-specific mortality model

^c^Statistical heterogeneity by race and ethnicity was assessed via an interaction term with continuous coffee intake and race/ethnicity using wald chi-square tests

Analysis of the association of caffeine intake from any source with risk of all-cause and CRC-specific mortality is shown in Table 4. The top quartile of caffeine intake, compared to the lowest quartile of caffeine intake, was associated with lower risk of all-cause mortality (HR 0.78 [0.62—0.99]) and CRC-specific mortality (HR 0.48 [0.27—0.87]).

**Table 4:** Association of post-diagnosis caffeine intake with risk of all-cause and CRC-specific mortality

	Caffeine intake by quartile					Per 1 SD increase/day (96 mg/day)
	1st	2nd	3rd	4th	*p*-trend	
*n*	274	275	275	274		1098
Median caffeine intake (mg/day) by quartile	12.05	66.88	108.30	235.58		93.51
*All-cause mortality*						
Events/person-years	170/2606	166/2792	151/2967	141/3022		628/11,387
Model 1 HR [95% CI]^a^	1 (ref.)	0.89 (0.72—1.11)	0.81 (0.65—1.02)	0.73 (0.58—0.92)	0.007	0.93 (0.86—1.01)
Model 2 HR [95% CI]^b^	1 (ref.)	0.96 (0.77—1.20)	0.91 (0.72—1.14)	0.78 (0.62—0.99)	0.028	0.95 (0.88—1.04)
*CRC mortality*						
Events/person-years	35/2606	24/2792	26/2967	22/3022		107/11,387
Model 1 HR [95% CI]^a^	1 (ref.)	0.64 (0.38—1.09)	0.67 (0.40—1.12)	0.59 (0.34—1.01)	0.093	0.90 (0.74—1.09)
Model 2 HR [95% CI]^b^	1 (ref.)	0.58 (0.34—1.01)	0.73 (0.43—1.25)	0.48 (0.27—0.87)	0.036	0.84 (0.68—1.04)

^a^Model 1 adjusts for age, sex, race and ethnicity, BMI, and years from diagnosis to survey completion

^b^Model 2 adjusted for factors in Model 1 and cancer site (colon vs rectum), stage, educational attainment, marital status, smoking, physical activity (< 60 min/week, 60–150 min/week, ≥ 150 min per week), comorbidity index, other cancer (dichotomous), alcohol intake (g/day), and omega 3 fatty acid intake (g/day)

Lastly, the association of change in coffee consumption (from pre-CRC diagnosis to post-CRC diagnosis) with risk of mortality is detailed in Table 5. Overall, increasing coffee intake was associated with lower risk of all-cause mortality, independent of coffee intake prior to diagnosis (HR per 1 cup increase/day: 0.89 [0.81–0.98]).

**Table 5:** Association of change in total coffee consumption pre- to post-CRC diagnosis with risk of mortality

	Maintain <1 cups/day	Decrease from ≥ 1 cups cups/day to <1 cups/day	Increase from <1 cups/day to ≥ 1 cups cups/day	Maintain ≥ 1 cups/day	Change in coffee intake per 1-cup increase/day^c^
*n*	273	200	85	521	1079^d^
*All-cause mortality*					
Events/person-years	159/2810	123/1923	48/890	286/5573	616/11,196
Model 1 HR [95% CI]^a^	1 (ref.)	1.18 (0.93—1.50)	0.95 (0.69—1.31)	0.89 (0.73—1.08)	0.90 (0.82—0.99)
Model 2 HR [95% CI]^b^	1 (ref.)	1.20 (0.94—1.53)	0.86 (0.62—1.20)	0.86 (0.70—1.06)	0.89 (0.81—0.98)
*CRC-specific mortality*					
Events/person-years	27/2810	20/1923	5/890	51/5573	103/11,196
Model 1 HR [95% CI]^a^	1 (ref.)	1.08 (0.60—1.95)	0.66 (0.25—1.72)	0.91 (0.56—1.46)	0.87 (0.69—1.10)
Model 2 HR [95% CI]^b^	1 (ref.)	0.91 (0.49—1.69)	0.60 (0.22—1.61)	0.83 (0.49—1.41)	0.84 (0.66—1.07)

^a^Model 1 adjusted for age, sex, race and ethnicity, BMI, and years from diagnosis to survey completion

^b^Model 2 adjusted for factors in Model 1 and cancer site (colon vs rectum), stage, educational attainment, marital status, smoking, physical activity (< 60 min/week, 60–150 min/week, ≥ 150 min per week), comorbidity index (detailed above in Table 1), other cancer (dichotomous), alcohol intake (g/day), and omega 3 fatty acid intake (g/day)

^c^Continuous models additionally adjusted for pre-CRC diagnosis total coffee intake (caffeinated and decaffeinated)

^d^19 participants were excluded because they were missing the initial FFQ data

The sensitivity analyses excluding participants with early mortality, distant disease, different treatment modalities, or with missing covariate data (as detailed in the methods) did not yield meaningfully different results.

## Discussion

In this prospective cohort study among individuals with CRC, there were no statistically significant associations between total or caffeinated coffee intake and risk of all-cause or CRC-specific mortality among the overall cohort. However, higher decaffeinated coffee intake was associated with lower risk of all-cause mortality. When this association was assessed by racial and ethnic group, the association remained statistically significant among the White participants. Additionally, among the White participants, higher intake of total coffee and caffeinated coffee were both associated with lower risk of CRC-specific mortality. Among the Native Hawaiian participants, higher total and caffeinated coffee were associated with lower risk of all-cause mortality. Further analysis revealed that high levels of caffeine intake were associated with lower risk of all-cause and CRC-specific mortality and increasing total coffee intake from pre-CRC diagnosis to post-CRC diagnosis was associated with lower risk of all-cause mortality. This study, conducted with a racially and ethnically diverse cohort, adds to the growing body of research drawing connections between coffee intake and risk of mortality among individuals with CRC.

Coffee has been suggested to impact CRC mortality through its antiproliferative, pro-apoptotic, antioxidant, anti-inflammatory, and insulin-sensitivity promoting effects [3–7]. There is growing evidence to support an association of coffee intake with lower risk of CRC-specific and all-cause mortality [8–13]. However, there is some data to support a non-linear association between coffee intake and CRC mortality; Oyelere et al. found a U-shaped association of coffee consumption and mortality with the lowest risk of all-cause mortality seen among participants consuming 4 cups/day [14]. Existing data also supports that caffeinated coffee consumption is associated with CRC disease-free survival and overall survival [8–10]. Data specifically on the impact of decaffeinated coffee on CRC is mixed; multiple studies have found associations between higher decaffeinated coffee intake and reduced CRC cancer risk, CRC-specific mortality, and all-cause mortality. [9, 10, 27] However, other work has found a null association between its consumption and CRC disease free and overall survival [8].

It was unexpected to observe an association for only decaffeinated coffee intake and risk of all-cause mortality, yet also observe that caffeine was associated with lower risk of all-cause and CRC-specific mortality. A possible explanation for these potentially conflicting results is that the participants in the MEC reported lower coffee consumption compared to prior cohorts, thus precluding analysis of the impact of coffee at the extreme of high consumption (≥ 4 cups per day) as done in other studies [8, 9, 14]. Our results for the association between higher total coffee intake and all-cause mortality did border on statistically significant (HR per 1 cup/day increase: 0.92 [0.85–1.00], *p*-value 0.057), suggesting that possibly with more extremes of coffee intake this result would have been significant. The lack of extremes may have been ameliorated in the caffeine analysis since it considers caffeine from other sources.

Our finding among only White participants that higher total and caffeinated coffee were associated with lower risk of CRC-specific mortality is concordant with the prior literature, which were conducted among primarily among White individuals. Our finding that higher total and caffeinated coffee were associated with lower risk of all-cause mortality among the Native Hawaiian group is similar to what prior literature has reported among overall study populations, however, prior literature has not specifically examined this ethnic group. Unlike the prior large prospective studies, our study is the first to include a racially and ethnically diverse sample [8, 9, 14]. The different results by racial and ethnic groups may reflect differences in levels of coffee intake (given the White participants had the highest reported total coffee and caffeine intake in our study population) or differences in coffee strength or additives that may affect the results. Additionally, there may be interactions between different culturally concordant diets and coffee consumption that may impact the gut microbiome or other epigenetic factors that ultimately impact risk of all-cause or CRC specific mortality. These disparate observations between the racial and ethnic groups highlight the importance of further research to examine the associations of coffee, and other dietary factors, with risk of mortality among more racially and ethnically diverse cohorts.

Our findings suggest that caffeine consumption specifically may be a protective factor against all-cause and CRC-specific mortality, regardless of the source of caffeine. This contributes to mixed prior literature. Guercio et al. found that greater caffeine intake was associated with longer disease-free survival among individuals with CRC but did not find this association with overall survival [8]. Conversely, Mackintosh et al. examined caffeine as a covariate in models examining the impact of coffee on overall survival and reported no significant association of caffeine intake [10]. Proposed mechanisms for the positive association between caffeine and CRC survival include that caffeine increases insulin sensitivity, which has been implicated in reducing CRC mortality, [6, 10, 28, 29] and provides anti-inflammatory properties inhibiting carcinogenesis [5]. If caffeine is partially driving the effect of coffee on CRC mortality, we hypothesize that the range of caffeine intake among our participants reporting caffeinated coffee intake only was not high enough to detect the impact on CRC mortality. Our data suggest that greater investigation should focus on the impact of caffeine specifically on CRC mortality, rather than coffee alone.

In our analysis, we found a significant association between increasing coffee intake and lower risk of all-cause mortality when examining change in coffee intake from pre- to post-diagnosis CRC as a continuous variable (while controlling for pre-diagnosis coffee intake). When examining change in coffee intake categorically, however, we found no significant association in relation to risk of all-cause or CRC-specific mortality. The significance within the continuous model may be driven by outliers given that for the categorical analysis, we were only able to examine an upper category of ≥1 cup/day due the distribution of coffee consumption. These findings add to existing work by Hu et al. that only examined categorical changes in coffee consumption and found no significant associations with increasing coffee consumption from <2 to ≥2 cups/day but did find lower CRC-specific and all-cause mortality among participants who maintained high consumption (≥ 2 cups) pre- to post-diagnosis [9].

This study has several limitations including a paucity of participants with high coffee intake among our sample, possibility limiting detection of differences in mortality seen among prior papers. However, critiques of prior studies examining coffee intake at the extremes have highlighted that individuals consuming extremes of coffee may represent more physically well patients and differences in mortality in those studies may reflect bias rather than true coffee effect. Thus, it may be more realistic and less prone to bias to examine the effect of more moderate coffee consumption. Second, although our study examines a more racially and ethnically diverse sample, there were small sample sizes of some racial and ethnic groups that limited individual group analysis. Third, the findings are susceptible to survival bias given the time between initial survey and follow-up survey in which participants needed to survive to be included in the analysis. Fourth, this is an observational study, and we cannot rule out the possibility of residual or unmeasured confounding. It is possible that the magnitude of confounding differs across our exposures of interest (for example, if people who consume decaffeinated coffee are more health conscious than those who consume caffeinated coffee). Fifth, based on prior studies, we comprehensively examined coffee-related exposures (e.g., total, caffeinated, decaffeinated) in relation to two primary outcomes; therefore, there is the possibility of observing statistically significant findings due to chance. Finally, we were unable to explore the impact of coffee intake on CRC recurrence given lack of access to this data. Future work should attempt to collect recurrence data and examine these questions among a larger, more racially and ethnically diverse sample population with a wider range of coffee consumption habits.

## Conclusions

In conclusion, among a multiracial, multiethnic group of individuals with CRC, total coffee and caffeinated coffee were associated with mortality risk in only some racial and ethnic groups. We also found that higher decaffeinated coffee consumption was associated with lower risk of all-cause mortality in the overall sample. Additional studies in more racially and ethnically diverse cohorts are needed to evaluate if these results are replicable and to increase our understanding of how coffee and caffeine intake may impact outcomes among patients with CRC.

## Supplementary Information

Below is the link to the electronic supplementary material.

Supplementary file1 (DOCX 51 KB)

## Data Availability

The data that support the findings of this study are available from the Multiethnic Cohort Study upon reasonable request and with permission of the Multiethnic Cohort Study through the online data request system (https://www.uhcancercenter.org/for-researchers/mec-data-sharing).

## Abbreviations

(CRC): Colorectal cancer
(HR): Hazard ratio
(CI): Confidence interval
(MEC): Multiethnic Cohort
(FFQ): Food frequency questionnaire
(SEER): Surveillance, epidemiology and end results
(BMI): Body mass index
(IQR): Interquartile range
(SD): Standard deviation
