# Supplementary material for: Association of coffee intake with risk of all-cause and colorectal cancer-specific mortality among individuals with colorectal cancer in the Multiethnic Cohort Study
Source: Cancer Causes Control. 2026 Sep 9;37(10):157. doi: 10.1007/s10552-026-02246-w (PMC13558410; doi:10.1007/s10552-026-02246-w)
Supplement: Supplementary file 1 [file 10552_2026_2246_MOESM1_ESM.docx]

**SUPPLEMENTAL TABLE**

| **Table S1.** Association between post-diagnosis total coffee plus cappuccino intake and risk of all-cause and colorectal cancer-specific mortality among 1,098 colorectal cancer survivors in the Multiethnic Cohort Study^a^ | | | | | | |
| --- | --- | --- | --- | --- | --- | --- |
|  | **Total coffee intake (cups per day)**^a^ | | | | | **Continuous,**  **per 1 cup/d** |
|  | **None** | **0 - <1** | **1 - <2** | **2+** | **P-trend** |  |
| **No. of participants** | 175 | 295 | 394 | 234 |  | 1098 |
| **All-cause mortality** | | | | | | |
| Events / person-years | 109 / 1710 | 171 / 3010 | 224 / 4131 | 124 / 2537 |  | 628 / 11387 |
| Model 1 HR [95% CI] ^b^ | 1 (ref.) | 0.96 (0.75, 1.22) | 0.83 (0.66, 1.05) | 0.82 (0.63, 1.07) | 0.10 | 0.94 (0.87, 1.02) |
| Model 2 HR [95% CI] ^c^ | 1 (ref.) | 1.04 (0.81, 1.34) | 0.85 (0.67, 1.08) | 0.82 (0.63, 1.08) | 0.06 | 0.92 (0.85, 1.00) |
| **CRC-specific mortality** | | | | | | |
| Events / person-years | 19 / 1710 | 28 / 3010 | 40 / 4131 | 20 / 2537 |  | 107 / 11387 |
| Model 1 HR [95% CI]^a^ | 1 (ref.) | 0.91 (0.50, 1.63) | 0.87 (0.50, 1.51) | 0.76 (0.40, 1.44) | 0.41 | 0.92 (0.76, 1.11) |
| Model 2 HR [95% CI]^b^ | 1 (ref.) | 1.02 (0.56, 1.87) | 0.98 (0.54, 1.78) | 0.81 (0.41, 1.61) | 0.45 | 0.89 (0.73, 1.08) |

^a^ Total coffee is the sum of regular coffee (brewed or instant), decaffeinated coffee (brewed or instant), and cappuccino (includes café au lait, caffé latte, café con leche).

^b^ Basic multivariable adjusted model adjusts for age, sex, race/ethnicity, BMI, and years from diagnosis to survey completion

^c^ Multivariable models adjusted for basic model factors (in Model 1) and cancer site (colon vs rectum), stage, educational attainment, marital status, smoking, physical activity (categorical), comorbidity index (see Table 1 for details), other cancers (dichotomous), alcohol intake (g/day), and omega-3 fatty acid intake (g/day)
